# Access to safe abortion: building choices for women living with HIV and AIDS

**DOI:** 10.1186/1758-2652-14-54

**Published:** 2011-11-14

**Authors:** Phyllis J Orner, Maria de Bruyn, Regina Maria Barbosa, Heather Boonstra, Jennifer Gatsi-Mallet, Diane D Cooper

**Affiliations:** 1 School of Public Health & Family Medicine, University of Cape Town, Cape Town, South Africa; 2 Ipas, Chapel Hill, North Carolina, USA; 3 Núcleo de Estudos de População, Universidade Estadual de Campinas, São Paulo, Brasil; 4 Guttmacher Institute, Washington DC, USA; 5 Namibia Women's Health Network, Windhoek, Namibia

## Abstract

In many areas of the world where HIV prevalence is high, rates of unintended pregnancy and unsafe abortion have also been shown to be high. Of all pregnancies worldwide in 2008, 41% were reported as unintended or unplanned, and approximately 50% of these ended in abortion. Of the estimated 21.6 million unsafe abortions occurring worldwide in 2008 (around one in 10 pregnancies), approximately 21.2 million occurred in developing countries, often due to restrictive abortion laws and leading to an estimated 47,000 maternal deaths and untold numbers of women who will suffer long-term health consequences. Despite this context, little research has focused on decisions about and experiences of women living with HIV with regard to terminating a pregnancy, although this should form part of comprehensive promotion of sexual and reproductive health rights.

In this paper, we explore the existing evidence related to global and country-specific barriers to safe abortion for all women, with an emphasis on research gaps around the right of women living with HIV to choose safe abortion services as an option for dealing with unwanted pregnancies. The main focus is on the situation for women living with HIV in Brazil, Namibia and South Africa as examples of three countries with different conditions regarding women's access to safe legal abortions: a very restrictive setting, a setting with several indications for legal abortion but non-implementation of the law, and a rather liberal setting.

Similarities and differences are discussed, and we further outline global and country-specific barriers to safe abortion for all women, ending with recommendations for policy makers and researchers.

## Review

Recently, there has been an overdue and important increase in research internationally into the sexual and reproductive intentions and human rights of women and men living with HIV [1-5]. Nevertheless, little research has focused on women living with HIV's (WLHIV's) decisions about and experiences with terminating a pregnancy, although this should form part of comprehensive promotion of reproductive health rights. Further, minimal research has been conducted on linking HIV services and abortion care, on unsafe abortion in the context of HIV, and consideration of which abortion methods may be most suitable for and acceptable to WLHIV [6-8]. Both HIV/AIDS and abortion are highly emotive and stigmatizing issues in many countries, often perpetuated and/or underscored by laws criminalizing HIV transmission and by restrictive abortion laws. An understanding of the context and factors that facilitate or hinder WLHIV's decisions and experiences regarding abortion is therefore of central importance to promoting this aspect of HIV-positive women's sexual and reproductive rights.

In this paper, we explore the existing evidence related to global and country-specific barriers to safe abortion. We emphasise research gaps around the rights of WLHIV to reproductive choice, including the right to safe abortion services. Based on published literature and anecdotal and/or unpublished data collected by the authors, we then examine WLHIV's access to public health sector safe abortion in Brazil, Namibia and South Africa as examples of three countries with different conditions regarding safe legal abortions.

We begin by providing data on unwanted pregnancies and abortion in the global context, as well as global and country-specific barriers to safe abortion care for all women. We then discuss reproductive choice issues affecting all women and WLHIV specifically, followed by a description of reported abortion access and experiences for women in a highly restrictive setting (Brazil), a country with legal provisions for abortion that are rarely honoured (Namibia), and a more liberal setting (South Africa).

### Unwanted pregnancy and unsafe abortion

In 2008, 41% of all pregnancies worldwide were reported as unintended or unplanned [9]. This exceptionally high level of unwanted pregnancy on a global scale results from many women's inability to make decisions within relationships on pregnancy-related intentions and decisions [10], and an unmet need for modern contraceptive methods [11]. In other words, while women may want to avoid pregnancy, they may inadvertently heighten their risk of unwanted pregnancies by using traditional, less effective contraceptive methods or no contraceptive method at all. For some women, this may be due to a belief that their risk of pregnancy is low. Some women are unable to afford modern methods or are unaware that they exist; other women do not know where to obtain modern methods or do not like their side effects. And many women face opposition, resistance or lack of support from their male partners to using contraceptives [9,10].

Approximately 50% of unintended pregnancies worldwide end in abortion, with 53% of those in developed countries (i.e., Australia, Europe, Japan, New Zealand, the United States and Canada) and 48% of those in developing countries (i.e., Africa, Latin America and the Caribbean, Asia excluding Japan, and Oceania excluding Australia and New Zealand) [9]. The reasons why millions of women undergo abortion, or if unable to access legal safe abortion, resort to unsafe means to end an unwanted pregnancy, vary. Some of the most common factors include: socio-economic hardship; a desire to postpone pregnancy to a more suitable time or stop childbearing altogether [12-14]; and women feeling that they have reached their optimal family size [15,16]. Additionally, women seek abortions due to pregnancy carrying social stigma in certain contexts, such as if a woman is considered too young or too old, still at school or if it occurs outside of marriage [6,7,13,15]. Women may also seek an abortion if the pregnancy is a result of rape or incest [17-19] and if the pregnancy occurs within an abusive or discordant couple relationship [6,7,12,13]. Low use of contraception or failed contraception, lack of access to appropriate sexual and reproductive health information and reluctance to attend a health service due to poor quality of care have also been reported as important factors underlying unintended pregnancy and hence abortion uptake [13,15,20].

#### Unsafe abortion

Unsafe abortion has a serious negative impact on women and on their health. Of an estimated 358,000 maternal deaths in 2008, 47,000 resulted from unsafe abortion complications, and untold numbers of women suffer long-term health consequences from abortion complications, such as infertility due to untreated infection [21]. Of the approximately 21.6 million unsafe abortions performed worldwide in 2008, 98% occurred in developing countries. In sub-Saharan Africa and in Latin America, unsafe abortion rates in 2008 were estimated at around 30 per 1,000 women aged 15-44 years [22]. Safe abortion care, as part of overall improvements in women's access to sexual and reproductive health care, can prevent nearly all these abortion-related maternal deaths and disabilities [23].

The incidence of unsafe abortion and maternal mortality from unsafe abortion is generally highest in countries with restrictive abortion legislation, which usually corresponds to developing countries [24]. However, a woman's probability of having an abortion is comparable whether she lives in a developed or a developing country [15]; the main difference lies in the safety of the abortion provided. For instance, in 2003, there were 26 abortions per 1,000 women aged 15-44 years in developed countries where almost all abortions are safe and legal, compared with 29 per 1,000 in developing countries with restrictive laws.

With regards to WLHIV, in many areas of the world where HIV prevalence is high and access to abortion is restricted (either by law or by social and cultural barriers, or both), rates of unintended pregnancy and unsafe abortion have also been shown to be high. For example, in Malawi where the HIV prevalence rate among adults aged 15-49 years was estimated at 11.9% in 2009 [25], unsafe abortions account for up to 30% of maternal deaths [26].

### Barriers to safe abortion

A host of factors constitute barriers to safe abortion for women generally, irrespective of HIV status. While some are associated with restrictive abortion laws and policies, others lie within the social realm and yet others are health service related.

#### Social factors

Socio-cultural and traditional norms regarding motherhood militate against abortion being seen as acceptable in societies [2,16,27,28]. These are underscored by inequitable gender relations, including socio-economic inequalities, and dominance of patriarchal ideology related to societal gender norms [6,7,17,29]. Other important barriers include women's systemic lack of resources in society that can lead to inability or delays in accessing abortion [13] and other sexual and reproductive health services. Acts of violence against healthcare providers and services providing abortions, and threats of intimidation and harm towards women seeking an abortion in some settings [15,27,30] also act as deterrents to women seeking safe abortions. Unsubstantiated pronouncements that having safe abortions are detrimental to women's mental and physical health also plays a role in discouraging abortion as an option for women faced with unintended pregnancies [28].

#### Health service-related factors

Insufficient safe abortion services or difficulties in accessing these services, even where they are legal, act as barriers to safe abortion for women. Access to reproductive services, including contraception and safe abortion services, were reported as inadequate in 55 developing countries, particularly in the countries' rural areas where most of the people live [31]. Poor, rural women are particularly disadvantaged in this regard by the high transport costs they incur and the length of time it takes to travel to the nearest health service providing abortions. In South Africa, despite very liberal legal provisions for abortion, provision of health services in general and of abortion services in particular is uneven across urban and rural areas. Ramkissoon *et al *reported that this unevenness contributed to why thousands of women in South Africa continued to die each year due to abortion-related complications from abortions performed by unskilled providers, and noted that the problem may be even bigger for WLHIV [32].

A further potentially powerful barrier to safe abortion in settings where abortion is legal is negative healthcare provider attitudes towards performing abortions and towards women who seek abortions. This often results in resistance or reluctance to perform or even assist in abortion procedures [33]. There is evidence that some healthcare providers in South Africa also refuse to undergo abortion training as they believe that once trained they will be forced to provide abortions [19]. Some healthcare providers in South Africa also discourage women from having abortions. For example, it has been reported that healthcare providers in the public health sector frequently act as "gatekeepers", discouraging or delaying women in obtaining abortions, refusing to provide any information about the procedure, or misinforming women about the legal conditions for abortion [6,7,33-36]. The contentious and complex nature of abortion is illustrated by the fact that when removed from the stigmatized service setting associated with inducing an abortion, some of these same providers are willing to care for women with incomplete abortions, perceiving this as fulfilling their professional duty [19].

The paucity of providers willing to provide abortions in settings where abortion is legal is a further barrier to provision of abortion services. In South Africa, conscientious objection by nurses and doctors reportedly hampers the ability of a significant proportion of facilities designated to provide abortion in providing these services. Ngwena argues that in this context:

The right to conscientious objection cannot be exercised ... to permit the health worker to impose anti-abortion views on the pregnant woman or society and vice-versa. The health worker has the freedom to choose to refuse to participate in abortion procedures ... however, the rights of the pregnant woman and the interests of society must be taken into account [37].

In addition, many healthcare workers who perform or assist in abortion care in South Africa face stigmatization within their working environment by other health professionals. This has led to some nurses leaving the services after only a short period of providing abortion care, further exacerbating the shortage of providers willing to provide such services [29].

### Living with HIV: women's (in)ability to exercise sexual and reproductive choice

The inequitable gender relations and sociocultural norms that underpin reproductive choice in many areas of the world, make it a particularly fraught situation for WLHIV attempting to balance their own needs against pro-natal social expectations on childbearing, on the one hand, and social disapproval and discrimination against PLHIV having children, on the other hand [4,6,7,38]. As Gogna *et al *point out:

[T]raditional gender roles and expectations and the social construction of sexuality are at the heart of the problem ... Reproductive challenges around people living with HIV show the persistence of gender inequalities. If sexuality and reproductive choices are often rendered invisible in the case of women ... this phenomenon is particularly acute for women living with HIV [39].

Other issues reported in the literature to have a bearing on WLHIV's reproductive decision making include: religious beliefs that militate against abortion acceptability; negative attitudes of peers, sexual partners and family members [6,7]; stigma associated with poverty and single motherhood [40]; and ambivalence towards a pregnancy, even if planned, among women and men living with HIV [41]. Moreover, violence may be the outcome for women who disclose their HIV status in different contexts, creating further difficulties in WLHIV's ability to make autonomous reproductive decisions.

#### Lack of adequate services for pregnancy prevention

Globally, sexual and reproductive health services and HIV-related services are usually offered separately [42]. For example, contraceptive services are primarily offered to married women and couples of reproductive age, while HIV-related services often target individuals at higher risk of HIV exposure. Ramkissoon *et al *reported that WLHIV in South Africa encountered numerous obstacles in preventing unwanted pregnancies, such as: lack of information on the most appropriate contraceptive methods; limited access to contraceptives in the postnatal period; minimal condom promotion for pregnant women despite the relatively high increased risk of becoming infected during pregnancy; and denying women access to sterilization services [32].

Situations such as this may be compounded and impact negatively on reproductive choices if WLHIV are faced with overt or covert discriminatory attitudes from healthcare providers [4,41,43]. People living with HIV (PLHIV) wanting children are frequently stigmatized, but accessing safe, legal abortions is nevertheless often problematic or highly restricted [6,7,44]. A study in Viet Nam suggested that health service providers contributed to placing WLHIV in a "double-bind" situation where motherhood is highly socially valued, yet was not encouraged in the case of WLHIV [38]. In Uganda, unintended pregnancy among women is high at 50%, but may be even greater among WLHIV [11]. In 2008, the US Centers for Disease Control and Prevention reported that among pregnant women on antiretroviral therapy in Uganda, 93% of the pregnancies were unintended. Yet access to legally induced abortion is highly restricted (allowed by law only if a woman's life is endangered) and abortion is often discouraged by healthcare providers, who do not consider WLHIV as qualifying for legal abortions on life endangerment grounds. For those WLHIV who want to terminate an unwanted pregnancy, many will seek an unsafe abortion due to the restrictive abortion law, and this can be dangerous due to risks of increased rates of infection and haemorrhage among WLHIV [11].

In South Africa, PLHIV have reported judgemental and discriminatory attitudes by healthcare workers regarding their reproductive intentions [45,46]. Moreover, some WLHIV in the KwaZulu-Natal Province reported being both actively dissuaded from accessing public health abortion services and afraid to ask for an abortion at these facilities [47]. They feared possibly being subjected to healthcare provider abuse if they had an abortion and doubted that they would get good abortion care. Additionally, some reported being told that they could have an abortion only if they agreed to be sterilized thereafter.

#### Factors associated with WLHIV's decisions to seek abortion

In the literature, decision making on abortion among WLHIV generally has been addressed in the context of wider investigation of sexual and reproductive health rights and services. Findings from several of these studies suggest that the likelihood of becoming pregnant and of seeking to terminate a pregnancy is similar regardless of HIV status [14,45,48]. Similar to other women, WLHIV seek to terminate unwanted pregnancies in spite of facing legal restrictions on abortion [47] and frequently lacking access to safe abortion services [14,49,50].

However, WLHIV may have unique reasons for wanting abortions. WLHIV seek abortions when they lack access to what they consider to be appropriate contraceptives in the context of HIV [51]. WLHIV who already have children [49,51], those in a more advanced stage of HIV or those in concordant couples relationships also report being more likely to seek abortions [52]. Other reasons include: fears that a continued pregnancy will compromise their health (e.g., when they have low CD4 counts or are suffering from opportunistic infections) [6,7]; fearing the possibility of infecting an infant; feelings that having another child may be a burden to other dependent children and family structures; and choosing to reserve resources to care for children they already have or for themselves and their partners [6,7,14,39,51].

### Building reproductive choice for WLHIV in Brazil, Namibia and South Africa

We now turn to a focus on issues pertaining to HIV and termination of pregnancy in Brazil, Namibia and South Africa. We start by outlining abortion law and related issues in the three countries.

#### Legal framework for abortion

Abortion in Brazil is legal only if pregnancy results from rape or if the pregnancy is considered life threatening for women [53]; however, HIV/AIDS is not considered life threatening. Despite this highly restrictive law, unsafe abortion is widely used by women in Brazil. In 2005, it was estimated that over one million unsafe abortions were performed, corresponding to an average rate of 2.07 unsafe abortions per 100 women between 15 and 49 years of age, or 30 unsafe abortions per 100 live births [54].

Correa has argued that "unsafe abortion is ... a major public health problem in Brazil", with many women ending up in hospital due to needless complications unlikely to have occurred if abortion was far less restrictive [53]. In 2004, it was reported that abortion complications accounted for 11.4% of maternal mortality [55]. Many women in Brazil resort to seeking post-abortion hospital care only in the case of severe complications. Women have reported being afraid to access post-abortion care because they fear that questions posed by health professionals about how the abortion occurred may place them at risk for subsequent arrest and imprisonment [56]. However, mortality from abortion complications is reported to be declining largely due to use of medication to induce abortion. Diniz *et al *reported that 50% of abortions were self-induced by women, with the majority of women using misoprostol to induce an abortion [57].

In Namibia, the law is less restrictive than in Brazil, but nevertheless has limited conditions under which legal abortion can occur. The Abortion and Sterilization Act (1975) provides that abortion is legal for rape, fetal malformation, danger to a woman's life, and for harm to a woman's physical and mental health. However, three physicians or psychiatrists are required to authorize that an abortion is necessary for these reasons. This makes having a legal abortion a cumbersome process in practice that can be discouraging for women seeking a legal abortion. In effect, women generally are not given information about their rights to legal abortion, and government public pronouncements refer to abortion as if it were illegal [J Gatsi-Mallet, personal communication, June 2010]. Pregnant women in Namibia reportedly avoid going to a hospital for abortions due to a widespread belief, often perpetuated by health professionals, that abortion is illegal and because no information is readily available regarding how to access legal abortions.

No official statistics are available for the number of abortions performed, but in 2005, it was reported that 20.7% of obstetric complications treated in public health facilities were abortion related [58]. In 2009, the Minister of Health and Social Services stated in an interview that illegal abortions remain a serious health problem in Namibia:

About one third of the [abortion-related] deaths were due to septic and illegally-induced abortion most likely unsafely performed somewhere ... Fifty-nine percent of the women dying of abortion-related complications were under the age of 25. This is consistent with other reports that increasingly young people resort to unsafe abortion or even commit suicide because of unwanted pregnancy [59].

In South Africa, women are afforded access to free, legal abortions within public health sector services. The Choice on Termination of Pregnancy (CTOP) Act (1996) [60] provides for legal abortion on request for all women, without age restrictions, up to 12 weeks gestation. After 12 weeks and up to 20 weeks, women can choose to have an abortion for health and socio-economic reasons on the recommendation of a midwife or medical practitioner. After 20 weeks, abortion is only legal due to severe fetal abnormalities or severe maternal physical or mental health disease. The 2004 CTOP Amendment Act [61] was promulgated to increase access to abortion services countrywide, particularly in rural areas, by easing the procedure for abortion facilities accreditation and allowing a wider spectrum of trained healthcare providers (e.g., registered nurses) to perform first-trimester abortions. The liberalization of conditions for legal abortion in South Africa has had a dramatic effect on mortality and morbidity resulting from abortion complications. These have declined by 91% and almost 50%, respectively [62]. The estimated total number of abortions performed in South Africa until April 2010 is 916,049. Despite all of this, however, many South African women continue to face numerous obstacles to safe abortions [6,7,13,32,63].

#### Dealing with unwanted pregnancies

Women in these three different countries, including WLHIV, face similar obstacles and constraints to preventing an unwanted pregnancy, most notably an inability to make autonomous sexual and reproductive choices. In Brazil, several factors underscore the reasons both for why unwanted pregnancies occur and why WLHIV seek abortions: underlying gender inequities, evident in poor dialogue between sexual partners; in the reluctance or even refusal to use a contraceptive by the male partner; difficulties in negotiating the terms of the sexual relationship; and lack of sexual and reproductive health services and rights [56].

In Namibia, anecdotal evidence suggests that both older and younger women are made vulnerable to unwanted pregnancies due to socio-economic dependence on male partners who refuse to use condoms, but also refuse to "allow" women to use other contraceptive methods [J Gatsi-Mallet, personal communication, June 2010]. Namibia's unemployment rate of 51.2% reportedly has hit young people especially hard, and many young women, dependent on their partners for any income they receive, lack the ability to convince partners not wanting to use male condoms to do so. WLHIV are also hampered by being offered a limited choice in contraceptive method, often being told by health professionals that only hormonal injectables are suitable for them. Young people are often refused contraceptives by health professionals who deem them too young to be sexually active; they sometimes also receive faulty information, as reported by some young WLHIV who were told that using contraceptives at an early age will make them infertile [J Gatsi-Mallet, personal communication, June 2010].

Similarly, in South Africa, WLHIV, like many other women in the country, have reported numerous interconnected reasons for unwanted pregnancies, including: an inability to negotiate condom use with male partners; irregular or non-use of contraceptives, sometimes due to fear of anticipated adverse side effects; health professionals refusing requests for sterilization; lack of money for transport to access contraceptive services; and, frequently, not knowing how the reproductive cycle works [6,7].

#### Reasons for seeking an abortion

In Brazil, a national-level study that explored the occurrence of induced abortion among WLHIV in 13 municipalities in five Brazilian regions and compared their socio-demographics with those of HIV-negative women showed that 13.3% of WLHIV had had induced abortions [64]. A convenience sample of 1,785 WLHIV attending STI/AIDS Reference Centres and 2,149 attending primary healthcare units and Women's Health Reference Centres responded to a structured self-administered questionnaire and deposited the questionnaire into an anonymous "ballot box". Independent correlates of lifetime induced abortion for both groups were: age, with older women reporting greater proportions of induced abortion; living in the poorest geographical region in the country (northern Brazil); age at sexual debut (up to 17 years); having had three or more lifetime sexual partners; having ever used intravenous drugs; and self-reporting that they had had a sexually transmitted infection. The results suggest that, in general, the characteristics of women who reported induced abortion in both groups were similar and that living with HIV appeared to have little specific effect on reproductive decision making of participants in the study [64].

Furthermore, results from a qualitative study in Brazil suggest that WLHIV, similarly to HIV-negative women, seek abortions due to difficulties in preventing unwanted pregnancies that are largely due to limited access to contraceptive methods, rather than due to HIV-positive status [65]. It also suggests that WLHIV not wanting to have children lack sexual and reproductive health services tailored to their specific needs, and as a result, are often compelled to resort either to tubal ligation or risk of an unintended pregnancy and having an unsafe abortion. It should be noted, however, that the neglect of women's sexual and reproductive health rights and related services, including the right to safe abortion, may be compounded in the case of WLHIV by the failure to address these broader issues within the AIDS movement in Brazil. This movement has tended to focus on the right of PLHIV to have children exclusively, rather than on women's right to choose either to have or avoid having children [R Barbosa, personal communication, June 2010].

In Namibia, WLHIV reported seeking an abortion due to concerns about worsening their health and fear of perinatal HIV transmission [J Gatsi-Mallet, personal communication, June 2010]. WLHIV in South Africa often sought abortions when they were unemployed and simultaneously not getting financial and/or emotional support from male partners or families, and hence unable to care for a child [6,7]. Some women reported that they did not want another child or that they were not ready to have a child. Others reported seeking an abortion because the pregnancy was due to rape or sexual coercion. While data suggests that WLHIV in South Africa faced disapproval if they became pregnant, they were concurrently unlikely to be supported by partners, family and the broader community in seeking an abortion, which remains highly stigmatized at a community and healthcare service level, regardless of HIV status and despite South Africa's liberalized abortion law [6,7,13].

#### Barriers to reproductive choice including safe abortion for WLHIV

WLHIV in Brazil and Namibia, and to a lesser extent South Africa, have limited access to appropriate sexual and reproductive health services, including access to a choice of contraceptive methods and adequate abortion services suitable to their needs. For instance, contraceptive methods other than male condoms are actively discouraged by health professionals in Brazil, often due to fears that condom use would decline, with negative effects on HIV prevention if other more effective methods to prevent pregnancy were encouraged in addition to condoms [66]. Nor is emergency contraception easily accessible to women, including WLHIV, which is also the case in Namibia [J Gatsi-Mallet, personal communication, June 2010] and in South Africa [67-69]. Women in Brazil are able to obtain emergency contraception from a gynaecologist, but many women reportedly refrain from doing this due to possible judgemental attitudes from physicians for not using condoms [66].

While some health professionals in Brazil and Namibia support WLHIV being able to have safe abortions, this has not translated into policy or improved access [56] [J Gatsi-Mallet, personal communication, June 2010]. Additionally, it has been reported that WLHIV in Brazil [70] and Namibia [43] have been coerced into having sterilizations when seeking abortions through the formal health service channels, making some women seek alternative, unsafe abortions.

WLHIV in Cape Town, South Africa reported being hampered in having an abortion due to difficulties in making autonomous sexual and reproductive health decisions within a context of strong social expectations that women should bear children [6,7]. Women reported having to contend with male partners' opposition to abortion; and many women also had to grapple with their own religious beliefs that deemed abortion as "murder". Some women feared that abortion would further harm their health (e.g., if loss of blood during the procedure resulted in decreased CD4 counts) [6,7]. Health service-related difficulties, similar to that hampering women's abortion access generally, also hindered WLHIV's ability to access safe abortions. These included health service providers acting as "gatekeepers" to access by discouraging abortion, often for religious or moral reasons, or misinforming women that they may have only one abortion [6,7].

Overall, there are notable similarities in abortion experiences for WLHIV in Brazil and Namibia where access to legal abortion is restrictive, but there is also some overlapping with many South African WLHIV's experiences of abortion, despite the different legal status of abortion in South Africa.

Restrictive legal barriers to safe abortion in Brazil and Namibia force women in general to resort to unsafe abortions and, although specific data on WLHIV's experiences is limited, it is likely that they would share similar experiences. As mentioned earlier, research in Brazil has shown that 50% of the women who reported having had at least one abortion during their lifetimes reported use of medical drugs to induce abortion [57]. Women living in urban areas of Brazil can purchase misoprostol, which is sold on the black market since it is legally restricted to hospital use only [71]. Similarly, university students in Namibia reportedly access information on the Internet about misoprostol, and thereafter buy it at local pharmacies to terminate an unwanted pregnancy [J Gatsi-Mallet, personal communication, June 2010]. However, in both settings, information on correct dosage and use is lacking, which may be particularly harmful for WLHIV's health. One way that women in both countries reportedly approach this problem is to share information on ways to perform clandestine abortions with their peers (e.g., with pharmaceutical drugs or possibly other "concoctions").

In South Africa, WLHIV's experiences of abortion underscore the complex and contested nature of abortion for all women in the country. WLHIV reported both positive and negative abortion experiences, with some women reporting that providers were helpful and compassionate and others reporting that they found them to be rude, hectoring and abusive, and that they provided inappropriate or misleading pre-abortion counselling [6,7]. Negative and mixed messages were common among healthcare workers who were uncomfortable in providing abortions or assisting in abortion provision. For instance, women were told that they could "do abortion, but don't come again". One woman reported that a provider informed her that she had a right to abortion, but was then told by the same provider that "you are murdering because this is a human being". Quality of care was also seen as substandard in some instances; for example, women reported that they aborted the products of conception or a fetus while sitting on a chair in the waiting room, and that staff in some settings refused to replace linen savers that were saturated with blood clots. Some women complained about being given hormonal injectables post-abortion without appropriate counselling or prior consent [6,7].

Other WLHIV in South Africa reported positive abortion experiences, including that abortion providers were welcoming, helpful and professional in their approach. One woman described her abortion providers as "very cool, very generous" [6]. As disclosure of HIV status is not mandatory to obtain an abortion in South Africa, the HIV status of WLHIV seeking abortions may not be known by a provider and WLHIV would theoretically receive the same treatment and care as other women. Practical experience seems to bear this out, even when a woman's HIV status was known to providers. Respondents in two studies in South Africa who disclosed HIV-positive status to providers or thought the providers knew their status reported feeling no discrimination towards them on that basis [6,7].

## Conclusions

Our exploration of the situation for WLHIV in accessing safe abortion care in Brazil, Namibia and South Africa shows that, as for women more generally in these three countries and elsewhere, comprehensive and appropriate sexual and reproductive choice and rights, care and treatment has not yet been achieved. In this regard, recommendations for further research on HIV and abortion would include to:

1) Determine whether there are differences in the abortion intentions of WLHIV who are either on or are not receiving antiretroviral therapy (ART).

2) Determine the prevalence and effects of unsafe abortions in WLHIV.

3) Determine whether different abortion methods require specific attention in order to be tailored to the specific needs of WLHIV, both those on and not yet on ART.

4) Determine how sexual and reproductive health services, including those for abortion and post-abortion care, can best be linked to/integrated with HIV care services in these varying country contexts.

5) Determine what information WLHIV would like regarding all their sexual and reproductive health options during counselling to meet their dual needs for safer pregnancy, as well as pregnancy prevention and termination, should an unintended pregnancy occur.

6) Determine in more detail, in each country, the specific barriers to safe abortion for WLHIV and recommend policies to overcome these.

In addition to the specific factors that pose unique difficulties for WLHIV wishing to have an abortion, it is imperative to address the broader context of ensuring the sexual and reproductive rights and choices of all women. Many countries already have laws permitting safe legal abortions for preserving a woman's physical and mental health and in cases of rape, incest and fetal malformation. However, restrictive abortion laws are an unacceptable infringement of women's human rights and of medical ethics, and decisive steps need to be taken to ensure that access to legal and safe abortion is available and obtainable to all women in need, including WLHIV. It is important in countries where abortion laws are restrictive, such as in Brazil and Namibia, to advocate and lobby for changes to the law in order to ease women's access to safe abortion. Liberalization of abortion law in South Africa was critical in making a difference to women's ability to access safe abortion.

Nevertheless, as the experience of South Africa shows, changing laws is not enough. It is equally important to work towards changing other socio-economic, gender and health service implementation factors that still make access to safe abortions difficult or impossible for many women. Abortion policy regulations should intentionally facilitate access to safe abortion services for all women, inform healthcare providers of their obligations in this regard, and inform women and men about the services to which they have a right. Action is needed by researchers, policy makers and programme and/or service implementers to create an environment in which all women and girls, including those living with HIV, can make sexual and reproductive health decisions with unhindered freedom, and are then enabled to carry out whatever decisions they make without coercion and in a safe manner. This would necessarily include expanding access to effective modern contraceptive methods and improving the quality and coverage of post-abortion care.

## Competing interests

The authors declare that they have no competing interests.

## Authors' contributions

PO drafted the manuscript. MdB, RB, HB, JGM and DC reviewed the drafts and gave comments. All authors have read and approved the final version of this manuscript.

## References

[B1] Kaida A Laher F Strathdee SA Janssen PA Money D Hogg RS Gray G Childbearing intentions of HIV-positive women of reproductive age in Soweto, South Africa: The influence of expanding access to HAART in an HIV hyperendemic setting Am J Public Health 2010 14 2 350 358 20403884 10.2105/AJPH.2009.177469 PMC3020203

[B2] Barbosa RM Aquino EML Heilborn ML Berquó ES Evaluation in sexual and reproductive health Cad. Saúde Pública 2009 14 Suppl 2 190 191 10.1590/s0102-311x2009001400001 19684926

[B3] Cooper D Moodley J Zweigenthal V Bekker LG Shah I Myer L Fertility intentions and reproductive health care needs of people living with HIV in Cape Town, South Africa: Implications for integrating reproductive health and HIV care services AIDS Beh 2009 14 Suppl 1 38 46 10.1007/s10461-009-9550-1 19343492

[B4] London L Orner P Myer L "Even if you're positive, you still have rights because you are a person': Human rights and the reproductive choice of HIV-positive persons Developing World Bioeth 2007 14 1 11 22 10.1111/j.1471-8847.2007.00223.x 18302539

[B5] Gruskin S Ferguson L O'Malley J Ensuring sexual and reproductive health for people living with HIV: An overview of key human rights, policy and health systems issues Reprod Health Matters 2007 14 29 Suppl 4 26 17531746 10.1016/S0968-8080(07)29028-7

[B6] Orner P de Bruyn M Harries J Cooper D A qualitative exploration of HIV-positive pregnant women's decision-making regarding abortion in Cape Town, South Africa Sahara J 2010 14 2 44 51 21409294 10.1080/17290376.2010.9724956

[B7] Orner P de Bruyn M Cooper D "It hurts but I don't have a choice, I'm not working and I'm sick": Decisions and experiences regarding abortion of women living with HIV in Cape Town, South Africa Cult Health Sex 2011 14 7 781 795 10.1080/13691058.2011.577907 21656408

[B8] de Bruyn M Donta B, Lusti-Narasimhan M, Argarwal D, Van Look PFA, Chander PP Unwanted pregnancy and abortion. What is the situation for HIV-positive women today? Strengthening Linkages between Sexual and Reproductive Health and HIV/AIDS 2007 Mumbai: Indian Society for the Study of Reproduction and Fertility/World Health Organization 15 39

[B9] Singh S Wulf D Hussain R Bankole A Sedgh G Abortion Worldwide: A Decade of Uneven Progress 2009 New York: Guttmacher Institute

[B10] Malarcher S Olson LG Hearst N Blas E, Kurup AS Unintended pregnancy and pregnancy outcome: Equity and social determinants Equity, Social Determinants and Public Health Programmes 2010 Geneva, WHO 177 197

[B11] The Alan Guttmacher Institute (AGI)-World Health Organization (WHO) Facts on induced abortion worldwide 2011 http://www.guttmacher.org/pubs/fb_IAW.pdf

[B12] Curtis C Meeting health care needs of women experiencing complications of miscarriage and unsafe abortion: USAID's postabortion care program J Midwifery Womens Health 2007 14 4 368 375 10.1016/j.jmwh.2007.03.005 17603959

[B13] Harries J Orner P Gabriel M Mitchell E Delays in seeking an abortion until the second trimester: a qualitative study in South Africa BMC Reprod Health 2007 14 7 10.1186/1742-4755-4-7 PMC2039724 17883835

[B14] Berer M HIV/AIDS, sexual and reproductive health: Intersections and implications for national programmes Health Policy Plan 2004 14 Suppl 1 162 170 10.1093/heapol/czh046 15452016

[B15] Shah I Åhman E Unsafe abortion: global and regional incidence, trends, consequences, and challenges J Obstet Gynaecol 2009 14 12 1149 1158 20085681

[B16] Oye-Adeniran B A Adewole IF Umoh AV Fapohunda OR Iwere N Characteristics of abortion care seekers in South-Western Nigeria Afr J Reprod Health 2004 14 3 81 91 10.2307/3583395 17348327

[B17] Ashenafi M Advocacy for legal reform for safe abortion Afr J Reprod Health 2004 14 1 79 84 10.2307/3583310 15487618

[B18] Braam T Hessini L The power dynamics perpetuating unsafe abortion in Africa: A feminist perspective Afr J Reprod Health 2004 14 1 43 51 10.2307/3583304 15487612

[B19] Harrison A Montgomery ET Lurie M Barriers to implementing South Africa's Termination of Pregnancy Act in rural KwaZulu/Natal Health Policy Plan 2000 14 4 424 431 10.1093/heapol/15.4.424 11124246

[B20] Meuwissen LE Gorter AC Knottnerus JA Perceived quality of reproductive care for girls in a competitive voucher programme. A quasi-experimental intervention study, Managua, Nicaragua Int J Qual Health Care 2006 14 1 35 42 10.1093/intqhc/mzi073 16421187

[B21] Shah I Åhman E Unsafe abortion in 2008: Global and regional levels and trends Repro Health Matters 2010 14 36 90 101 10.1016/S0968-8080(10)36537-2 21111353

[B22] World Health Organization (WHO) Unsafe Abortion: Global and Regional Estimates of Incidence of Unsafe Abortion and Associated Mortality in 2008. Sixth Edition 2011 Geneva: WHO

[B23] World Health Organization (WHO) Packages of Interventions for Family Planning, Safe Abortion Care, Maternal, Newborn and Child Health 2010 Geneva: WHO

[B24] Fawcus SR Maternal mortality and unsafe abortion Best Pract Res Clin Obstet Gynaecol 2008 14 3 533 548 10.1016/j.bpobgyn.2007.10.006 18249585

[B25] UNAIDS Uniting the world against AIDS 2010 http://www.unaids.org/en/CountryResponses/Countries/malawi.asp

[B26] Semu-Banda P Women's group sues government over abortion rights 2011 http://ipsnews.net/africa/nota.asp?idnews=46671

[B27] McGovern T Building coalitions to support women's health and rights in the United States: South Carolina and Florida Reprod Health Matters 2007 14 29 119 129 10.1016/S0968-8080(07)29290-0 17512383

[B28] Hessini L Global progress in abortion advocacy and policy: An assessment of the decade since ICPD Reprod Health Matters 2005 14 25 88 100 10.1016/S0968-8080(05)25168-6 16035601

[B29] Mhlanga RE Abortion: Developments and impact in South Africa Br Med Bull 2003 14 1 115 126 10.1093/bmb/ldg006 14711758

[B30] de Bruyn M Living with HIV: Challenges in reproductive health care in South Africa Afr J Reprod Health 2004 14 1 92 98 10.2307/3583312 15487620

[B31] Ross JA Begala JE Measures of strength for maternal health programs in 55 developing countries: The MNPI study Matern Child Health J 2005 14 Suppl 29 59 70 15880975 10.1007/s10995-005-2548-z

[B32] Ramkissoon AH Coovadia J Hlazo A Coutsoudis A Mthembu P Smit J Ijumba P, Padarath A Options for HIV-positive women African Health Review, Chapter 19 2006 Pretoria: Health Systems Trust 315 332

[B33] Harries J Stinson K Orner P Health care providers' attitudes towards termination of pregnancy: A qualitative study in South Africa BMC Public Health 2009 14 296 10.1186/1471-2458-9-296 19689791 PMC2734857

[B34] Hall W Roberts J Understanding the Impact of Decentralisation on Reproductive Health Services in Africa: South Africa Report 2006 Durban: Health Systems Trust

[B35] Wood K Jewkes R Blood blockages and scolding nurses: Barriers to adolescent contraceptive use in South Africa Reprod Health Matters 2006 14 27 109 118 10.1016/S0968-8080(06)27231-8 16713885

[B36] Cooper D Morroni C Orner P Moodley J Harries J Cullingworth L Hoffman M Ten years of democracy in South Africa: Documenting transformation in reproductive health policy and status Reprod Health Matters 2004 14 24 70 85 10.1016/S0968-8080(04)24143-X 15626198

[B37] Ngwena C Conscientious objection and legal abortion in South Africa: delineating the parameters J Juridical Sci 2003 14 1 1 18

[B38] Chi BK Hanh N Rasch V Gammeltoft T Induced abortion among HIV-positive women in Northern Vietnam: Exploring reproductive dilemmas Cult Health Sex 2010 14 Suppl 1 41 54 2009 10.1080/13691050903056069 19588275

[B39] Gogna ML Pecheny MM Ibarlucίa I Manzelli SBL The reproductive needs and rights of people living with HIV in Argentina: Health service users' and providers' perspectives Soc Sci Med 2009 14 6 813 820 19577833 10.1016/j.socscimed.2009.06.002

[B40] Kirshenbaum SB Hirky AE Correale J Goldstein RB Johnson MO Rotheram-Borus MJ Ehrhardt A 'Throwing the dice': Pregnancy decision-making among HIV-positive women in four US cities Perspect Sex Reprod Health 2004 14 3 106 112 10.1363/3610604 15306272

[B41] Sable MR Libbus MK Jackson D Hausler H The role of pregnancy intentions in HIV prevention in South Africa: a proposed model for policy and practice Afri J AIDS Res 2008 14 2 159 165 10.2989/AJAR.2008.7.2.1.518 25864392

[B42] Maynard-Tucker G HIV/AIDS and family planning services integration: review of prospects for a comprehensive approach in sub-Saharan African Afri J AIDS Res 2009 14 4 465 472 10.2989/AJAR.2009.8.4.10.1047 25875710

[B43] International Community of Women Living with HIV/AIDS (ICW) Namibian government called on to respond to the alleged sterilization of women living with HIV/AIDS 2009 London: ICW

[B44] Segurado AC Paiva V Rights of HIV positive people to sexual and reproductive health: Parenthood Reprod Health Matters 2007 14 Suppl 29 27 45 17531747 10.1016/S0968-8080(07)29032-9

[B45] Cooper D Harries J Myer L Orner P Bracken H Zweigenthal V "Life is still going on": Reproductive intentions among HIV-positive women and men in South Africa Soc Sci Med 2007 14 2 274 283 10.1016/j.socscimed.2007.03.019 17451852

[B46] Meel BL Ethical issues related to HIV/AIDS: Case reports J Clin Forensic Med 2005 14 3 149 152 10.1016/j.jcfm.2004.10.018 15914310

[B47] de Bruyn M Women, reproductive rights, and HIV/AIDS: Issues on which research and interventions are still needed J Health Popul Nutr 2006 14 4 413 425 17591338 PMC3001145

[B48] Hebling EM Hardy E Feelings related to motherhood among women living with HIV in Brazil: A qualitative study AIDS Care 2007 14 9 1095 1100 10.1080/09540120701294294 18058393

[B49] de Bruyn M Safe abortion for HIV-positive women with unwanted pregnancy: A reproductive right Reprod Health Matters 2003 14 22 152 161 10.1016/S0968-8080(03)02297-3 14708406

[B50] Feldman R Maposhere C Safer sex and reproductive choice: Findings from "positive women: voices and choices" in Zimbabwe Reprod Health Matters 2003 14 22 162 173 10.1016/S0968-8080(03)02284-5 14708407

[B51] International Community of Women Living with HIV/AIDS (ICW) Addressing the needs of HIV-positive women for safe abortion care 2008 London: ICW

[B52] Floridia M Tamburrini E Tibaldi C Anzidei G Muggiasca ML Meloni A Guerra B Maccabruni A Molinari A Spinillo A Dalzero S Ravizza M Italian Group on Surveillance on Antiretroviral Treatment in Pregnancy Voluntary pregnancy termination among women with HIV in the HAART era (2002-2008): A case series from a national study AIDS Care 2010 14 1 50 53 10.1080/09540120903033268 20390480

[B53] Correa S Brazil: One of the abortion front lines Reprod Health Matters 2010 14 36 111 117 10.1016/S0968-8080(10)36532-3 21111355

[B54] Adesse L Monteiro MFG Magnitude do aborto no Brasil: Aspectos Epidemiológicos e Sócio-Culturais 2007 http://www.ipas.org/Publications/asset_upload_file702_3556.pdf

[B55] Laurenti R Mello Jorge MHP Gotlieb SLD A mortalidade materna nas capitais brasileiras: algumas características e estimativa de um fator de ajuste Rev Bras Epidemiol 2004 14 4 449 460 10.1590/S1415-790X2004000400008

[B56] Menezes G Aquino EML Pesquisa sobre o aborto no Brasil: avanços e desafios para o campo da saúde coletiva Cad Saúde Pública 2009 14 Suppl 2 S193 S204 19684927 10.1590/s0102-311x2009001400002

[B57] Diniz D Medeiros M Aborto no Brasil: uma pesquisa domiciliar com técnica de urna Ciênc. saúde coletiva 2010 14 Suppl 959 966 10.1590/s1413-81232010000700002 20640252

[B58] World Health Organization (WHO) Maternal and child health in Namibia 2009 2 Geneva: WHO

[B59] Nyangove P Illegal abortions common despite risks 2009 http://ipsnews.net/africa/nota.asp?idnews=48759

[B60] Department of Health, Republic of SouthAfrica Choice on Termination of Pregnancy Act (CTOP) (No. 92 of 1996) 1996 Pretoria. Department of Health

[B61] Department of Health, Republic of South Africa The Choice on Termination of Pregnancy (CTOP) Amendment Act (No. 38 of 2004 2004 Pretoria: Department of Health

[B62] Gabriel M Legal abortions have saved thousands of women from death and suffering Cape Times 2008

[B63] Jewkes RK Gumede T Westaway MS Dickson K Brown H Rees H Why are women still aborting outside designated facilities in metropolitan South Africa? BJOG 2005 14 9 1236 1242 10.1111/j.1471-0528.2005.00697.x 16101602

[B64] Barbosa RM Pinho A Santos NJS Filipe E Villela W Aidar T Aborto induzido entre mulheres em idade reprodutiva vivendo e não vivendo com HIV/Aids no Brasil Ciênc Saúde Coletiva 2009 14 4 795 807 10.1590/s1413-81232009000400015 19721949

[B65] Villela WV Barbosa RM Felipe EV Portella AP Pantoja ALN Oliveira A Cardoso JS Induced abortion scenarios: are they different in the presence of HIV/AIDS? Proceedings of the XVIII International Aids Conference: 18-23 July 2010; Vienna

[B66] Barbosa RM Direitos reprodutivos e a transmissão vertical do HIV: 5 anos depois1o Encontro Paulista de Prevenção e Controle de DST/Aids 2009 14 São Paulo: Coordenação Estadual de DST/AIDS 19 23

[B67] Rogan M Nanda P Maharaj P Promoting and prioritizing reproductive health commodities: understanding the emergency contraception value chain in South Africa Afri J Reprod Health 2010 14 1 9 20 20695135

[B68] Myer L Mlobeli R Cooper D Smit J Morroni C Knowledge and use of emergency contraception among women in the Western Cape province of South Africa: a cross-sectional study BMC Women's Health 2007 14 14 17850659 10.1186/1472-6874-7-14 PMC2031876

[B69] Mqhayim MM Smit JA McFadyen ML Beksinska M Connolly C Zuma K Morroni C Missed opportunities: emergency contraception utilisation by young South African women Afri J Reprod Health 2004 14 2 137 44 10.2307/3583187 15623128

[B70] Hopkins K Barbosa RM Knauth DR Potter JE The impact of health care providers on female sterilization among HIV-positive women in Brazil Soc Sci Med 2005 14 3 541 554 10.1016/j.socscimed.2004.12.011 15899314

[B71] Diniz D Castro R The illegal market for gender-related drugs as portrayed in the Brazilian news media: the case of misoprostol and women Cad Saúde Pública 2011 14 1 94 102 21340108 10.1590/s0102-311x2011000100010

